# Pressure reconstruction method for spontaneous breathing effort monitoring

**DOI:** 10.1186/cc14339

**Published:** 2015-03-16

**Authors:** N Damanhuri, YS Chiew, NA Othman, PD Docherty, GM Shaw, JG Chase

**Affiliations:** 1 University of Canterbury, Christchurch, New Zealand; 2 Christchurch Hospital, Christchurch, New Zealand

## Introduction

Estimating respiratory mechanics of mechanically ventilated (MV) patients is unreliable when patients exhibit spontaneous breathing (SB) efforts on top of ventilator support. This reverse triggering effect [1] results in an M-wave shaped pressure wave. A model-based method to reconstruct the affected airway pressure curve is presented to enable estimation of the true underlying respiratory mechanics of these patients.

## Methods

**Figure 1:**
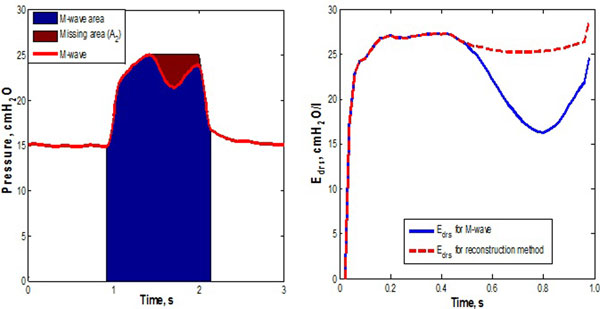
***E_drs_*for M-wave and reconstructed airway pressure at PEEP = 15 cmH_2_O**.

Airway pressure and flow data from 72 breaths of a pneumonia patient were used for proof of concept. A pressure wave reconstruction method fills parts of the missing area caused by SB efforts and reverse triggering by connecting the peak pressure and end-inspiration slope (Figure 1). A time-varying elastance model [2] was then used to identify underlying respiratory elastance (AUC*E_drs_*). The area of the unreconstructed M-wave has less pressure, resulting in a lower overall AUC*E_drs_*without reconstruction. The missing area of the airway pressure or AUC*E_drs_*is hypothesized to be a surrogate of patient-specific inspiratory to assess the strength of SB efforts. AUC*E_drs_*and missing area A_2_ are compared with/without reconstruction.

## Results

Median AUC*E_drs_*and breath-specific effort using reconstruction were 24.99 (IQR: 22.90 to 25.98) cmH_2_O/l and 3.64 (IQR: 0.00 to 3.87)% versus AUC*E_drs_*of 20.87 (IQR: 15.24 to 27.48) cmH_2_O/l for unreconstructed M-wave data, indicating significant patient and breath-specific SB effort, and the expected higher elastance (*P *< 0.05).

## Conclusion

A simple reconstruction method enables the real-time measurement of respiratory system properties of a SB patient and measures the surrogate of the SB effort, that latter of which has clinical use in deciding whether to extubate or re-sedate the patient.
